# Prognostic factors for overall survival in patients with spinal metastasis secondary to prostate cancer: *a systematic review and meta-analysis*

**DOI:** 10.1186/s12891-020-03412-0

**Published:** 2020-06-17

**Authors:** Zhong-yu Gao, Tao Zhang, Hui Zhang, Cheng-gang Pang, Wen-xue Jiang

**Affiliations:** 1 Department of Orthopedic Surgery, Tianjin First Central Hospital, 24 Fukang Rd, Nankai District, Tianjin, 300192 China; 2 Graduate School, Tianjin Medical University, Tianjin, 300070 China

**Keywords:** Spinal metastasis, Prognostic factors, Meta-analysis, Prostate cancer

## Abstract

**Background:**

To guide the selection of treatments for spinal metastases, the expected survival time is one of the most important determinants. Few scoring systems are fully applicable for spinal metastasis secondary to prostate cancer (PCa). This study aimed to identify the independent factors to predict the overall survival (OS) of patients with spinal metastases from PCa.

**Methods:**

The PubMed, Embase and CENTRAL were retrieved by two reviewers independently, to identify studies analyzed the prognostic effect of different factors in spinal metastasis from PCa. A systematic review and quantitative meta-analysis was conducted with hazard ratio (HR) and 95% confidence interval (95%CI) as the effect size.

**Results:**

A total of 12 retrospective cohort studies (1566 patients) were eligible for qualitative synthesis and 10 for quantitative meta-analyses. The OS was significantly influenced by performance status, visceral metastasis, ambulatory status and time from PCa diagnosis in more than half of the available studies. The meta-analyses demonstrated that OS was significantly influenced by visceral metastasis (HR = 2.24, 95%CI:1.53–3.27, *p* < 0.001), pre-treatment ambulatory status (HR = 2.64, 95%CI:1.82–3.83, *p* < 0.001), KPS (HR = 4.45, 95%CI:2.01–9.85, *p* < 0.001), ECOG (HR = 2.96, 95%CI:2.02–4.35, *p* < 0.001), extraspinal bone metastasis (HR = 2.04, 95%CI:1.13–3.68, *p* = 0.018), time developing motor deficit (HR = 1.57, 95%CI:1.30–1.88, *p* < 0.001) and time from PCa diagnosis (HR = 1.37, 95%CI:1.17–1.59, *p* < 0.001).

**Conclusions:**

Visceral metastasis, ambulatory status, extraspinal bone metastasis, performance status, time developing motor deficit and time interval from primary tumor diagnosis were significantly associated with the OS for spinal metastasis from PCa. When selecting the treatment modality, clinicians should fully consider the patients’ systematic status based on all potential prognostic factors.

**Level of evidence:**

I Meta-analysis.

## Background

Prostate cancer (PCa) is the most frequent malignant tumor and the second leading cause of cancer-related death in men from many developed countries [1]. PCa is generally sensitive to androgen blockade and chemotherapy, which can be divided into hormone-refractory type and hormone-naive type according to the response to hormone therapy. Though the prognosis of PCa is much better than that of many other malignant tumors such as lung cancer or gastrointestinal cancer, 80 to 100% of patients with advanced PCa develop bone metastases, of which the spine is the most common site of bone metastases [2, 3]. When spinal metastases occur, about one-third of patients will have symptoms, including pathological fractures, metastatic spinal cord compression (MSCC), and pain, which often severely impairs patients’ overall survival and quality of life [4, 5].

Many therapeutic modalities could be applied for patients with metastatic spinal disease from PCa. Generally, surgical intervention is required for vertebral compression fractures, vertebral segment instability, intractable pain and progressive neurological deficits, paraplegia, or quadriplegia [6, 7]. Unlike spinal metastases from some other cancers, such as renal cell carcinoma and malignant melanoma, metastatic spinal tumor from PCa are often more sensitive to radiation therapy (RT) [8, 9]. Thus, mild MSCC is also sensitive to external radiation therapy. In additional, for most newly diagnosed cases of spinal metastases from PCa, castration by reducing testosterone level though surgery or medication also has a significant effect on the relief of bone pain symptoms.

In the selection of treatment options for patients with spinal metastases, the patient’s expected survival time is one of the most important determinants. At present, in order to more accurately predict the survival time of patients and guide clinical treatment, many corresponding scoring systems have been proposed, including Tokuhashi [10], Tomita [11], Van der Linden [12], Bauer [13], and so on. These scoring systems contain some important prognostic factors that predict overall survival, such as patients’ performance status, number of extra-spinal or spinal bone metastases, visceral metastases, primary tumor type, and pathological fractures. However, the prognostic factors used in these scoring systems are not completely consistent, given contradictory survival expectations of specific patients based on different scoring systems. In addition, few scoring systems are fully applicable to specific primary tumor types. Several retrospective cohort studies have identified some potentially significant predictors for overall survival in patients with spinal metastasis from PCa [14–16]. However, these studies were conducted based on small samples, and different factors were included for analysis. Therefore, the purpose of this study is to identify the independent factors to predict the overall survival of patients with spinal metastases from PCa.

## Methods

### Data source and studies retrieval

This review was conducted according to the guidelines outlined in Preferred Reporting Items for Systematic Reviews and Meta-analysis (PRISMA) statement [17]. Two individual reviewers retrieved the platforms of PubMed, Embase and CENTRAL, from the inception to October 2019. The key words used for searching include “spinal metastasis”, “prostate cancer”, “overall survival” and “prognostic factor”. In additional, the reference lists of the included studies were screened and potentially related studies were hand-searched for possible inclusion.

### Inclusion and exclusion criteria

All retrieved records were screened for final inclusion based on the following inclusion criteria: (1) patients diagnosed as spinal metastasis from PCa; (2) studies associated with evaluating the prognostic effect of predict factors of overall survival; (3) studies designed as observational clinical study, including cohort studies and case-control studies would be eligible for inclusion. Studies would be excluded based on the following criteria: (1) duplicated studies; (2) animal studies, literature review, commentary studies and meta-analyses; (3) studies used the same cohort.

#### Study selection and data extraction

After excluding the duplicates, the remained records were screened with their titles /abstracts according to the inclusion criteria. Then, the potentially related titles /abstracts were further assessed for the final inclusion using their full texts.

Two authors independently extracted the following data from included studies:

1. Study characteristics: lead author, publication year, lead author’s country, study design and study period;
2. Patients information: numbers of involved patients and patients with MSCC, median age, pre- and post-treatment neurological status, performance status, visceral metastasis, extraspinal bone metastasis, number of involved vertebrae, distribution of involved vertebrae, prostate-specific antigen (PSA), Gleason grade of PCa, and hormonal status (hormone-refractory or hormone-naive PCa);
3. Treatment modalities: treatment of primary PCa, major treatment to spinal lesions, adjuvant therapies prior to and after major treatment, indication for surgery, re-operation and complications.
4. Outcomes information: overall survival and the associated prognostic factors.

We determined the cause of diversity in obtained information and resolved disagreement through discussion.

### Quality assessment of included studies

Two reviewers performed the process of quality assessment independently using the Newcastle-Ottawa Scale (NOS) [18]. This scale employs a 9 stars system that assesses three domains: patient selection, comparability of study groups and ascertainment of study outcome. Studies with a score of less than 6 indicates a high chance of bias.

#### Quantitative data analysis

As the prognostic effects of the factors were represented with hazard ratio (HR) and 95% confidence interval (95%CI) in the primary studies, meta-analysis was performed using HR as effect size. In case with significant heterogeneity (I^2^ > 50%, or *p* < 0.1 by Q test), random-effect model would be employed, while fixed-effect model was selected when no significant heterogeneity exists [19]. Z test was used to test the significance of the pooled effect size.

When five or more studies were included in a quantitative analysis, sensitivity analysis and publication bias test (Begg’s and Egger’s regression asymmetry test, *p* < 0.050 and *p* < 0.100 were considered to be with significant publication bias respectively) would be conducted [20].

The statistical procedures were conducted through software of Stata version 15.0 (StataCorp LLC, College Station, Texas, USA). The statistical significance was defined at a two-sided *p* value of less than 0.05.

## Results

### Study searching and selecting

**Fig. 1:**
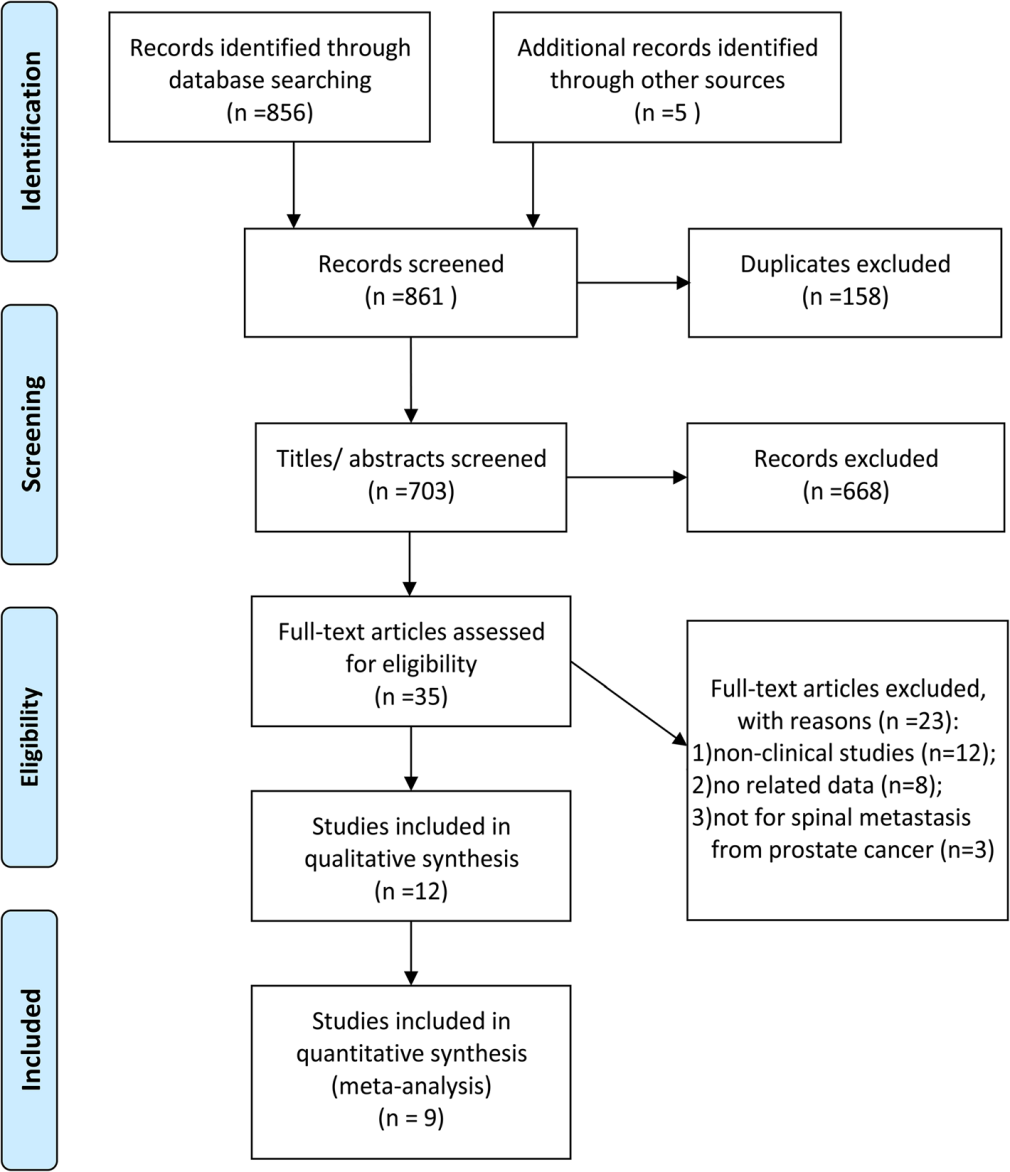
Flowchart of study retrieving and selecting

The flowchart of the study searching and selecting is shown in Fig. 1. A total of 861 records were screened in the initial searching. After excluding of 158 duplicates, 703 titles /abstracts were assessed for potential eligibility. Then, 35 full-text articles were further screened, remaining 12 eligible studies [14–16, 21–29] for qualitative synthesis and 10 eligible studies [14–16, 22, 23, 25–29] for quantitative meta-analysis finally.

### Characteristics of the included studies

**Fig. 2:**
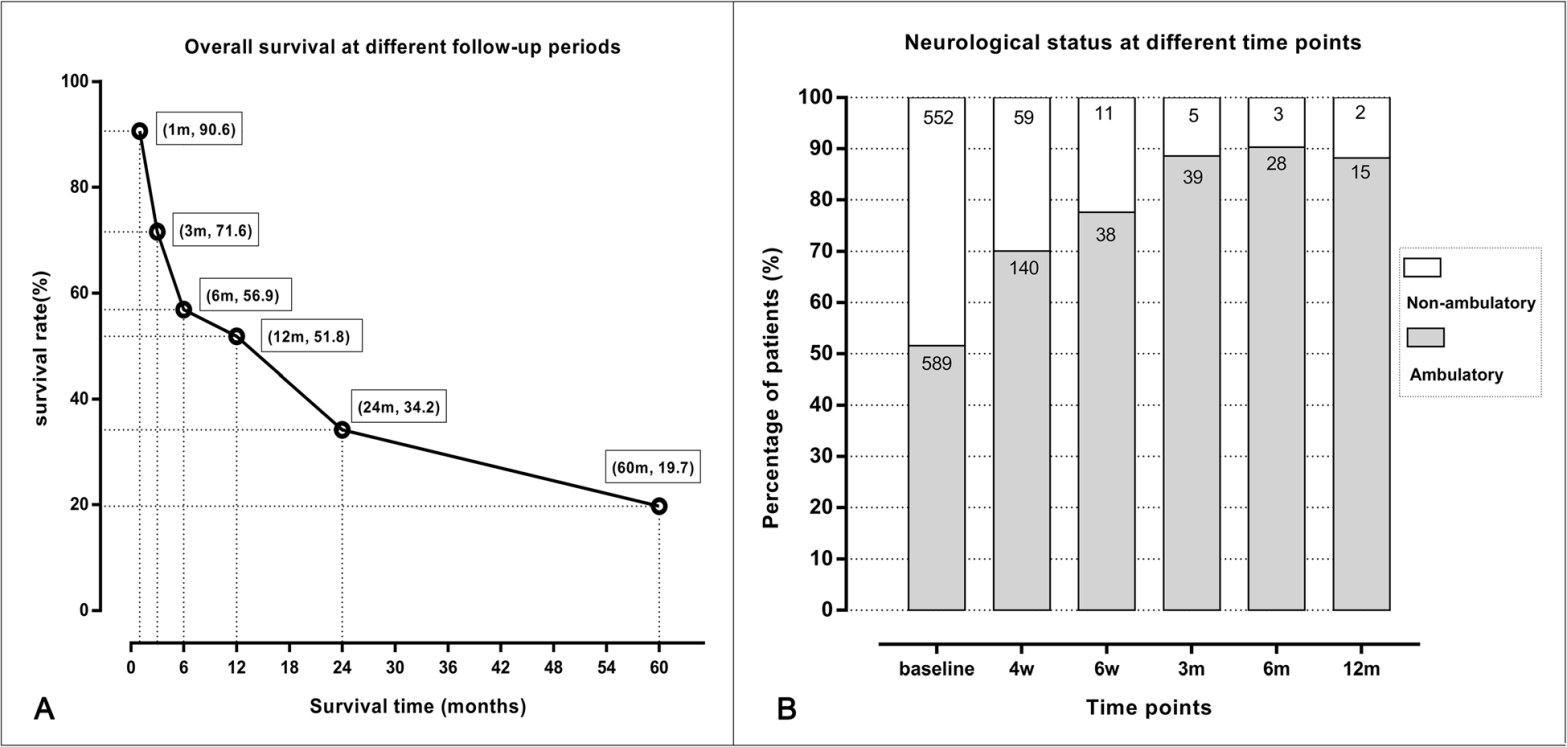
The overall survival rates at different follow-up periods (**a**), and the percentages of ambulatory and non-ambulatory patients at different time points (**b**). Numbers of related patients at different time points were presented in the bars of the part B

The summary of the included studies is available in Table 1. A total of 1566 patients were retrospectively included in these cohort studies, among which 1189 patients were proven to be with MSCC. The median ages ranged from 65 to 77 years among the included cohorts. The prevalence of visceral metastasis and extraspinal bone metastasis was available in nine [14–16, 22–25, 27, 29] and eight studies [15, 16, 22–25, 27, 28] respectively, with incidences of 26.7% (355 of 1329 patients) and 69.6% (864 of 1242 patients). The number of involved vertebrae was reported in seven studies [21, 23, 24, 26–29], giving 490 (46.1%) patients with 1–2 spinal lesions and 572 (53.9%) patients with 3 or more than 3 spinal lesions. The median overall survival was presented in eight studies with a range of 4–44 months. The overall survival rates at different follow-up periods are shown in Fig. 2a. The survival rates at 1, 3, 6, 12, 24 and 60 months after treatment were 90.6% (135/149 patients), 71.6% (161/225 patients), 56.9% (568/999 patients), 51.8% (691/1334 patients), 34.2% (66/193 patients) and 19.7% (6/29 patients) respectively. Figure 2b shows the percentages of ambulatory and non-ambulatory patients at different time points. Among the alive patients, the percentage of the ambulatory patients continues to increase.

**Table 1:** Summary of included studies

Study ID	Study period	Country	Patients (n)	Patients with MSCC	Age -median (range)	Distribution of involved vertebrae	PSA (ng/ml) -median (range)	Gleason score	Visceral met.	Other bone met.	Involved vertebrae -n	Performance status	Overall survival (median values & OS%)	hormone status	NOS
Ju, 2013 [15]	2002–2011	USA	27	27	65 (46–82)	C-1,CT-3,T-3,TL-7,L-1,LS-1,S-0,C/T/L-5,T/L/S-6	50 (0.1–11,900)	median: 8.5 (range, 6–10)	14	26	NA	KPS: 10–70:19; 80–100:8	1)10 (95%CI, 5–16)m; 2)OS%-1/3/6/9/24 m: 96/81/70/62/40	HR:24; HN:3	8
Meng, 2016 [16]	2002–2012	China	29	NA	71 (59–83)	C-5,T-11,L-12,S-1	NA	NA	7	10	NA	KPS: 10–70:26; 80–100:3	1)44 (range,1–80)m; 2)OS%-12/24/60 m: 89.7/74.4/19.7	HR:9; HN:20	7
Huddart, 1997 [21]	1984–1992	UK	69	69	NA	C-5,T-44,L/S-20	NA	NA	NA	NA	1:47; ≥2:20	NA	1)4 (range, 0.2–67)m; 2)OS%-24 m: 25	NA	8
Williams, 2009 [22]	1993–2005	USA	44	44	68 (51–85)	C-1,CT-1,T-3,TL-11,L-4,LS-1,S-3,C/T/L-15,T/L/S-5	27.5 (2.4–1520)	median: 8 (range, 2–10)	34	42	NA	NA	1)5 (95%CI, 1–10)m 2)NA	NA	9
Rades, 2012 [23]	1992–2010	Germany	436	436	NA	NA	NA	NA	100	252	1–2:178; ≥3:258	ECOG: 1–2:220; 3–4:216	1)NA 2)OS%-6/12 m: 57.3/45.0	NA	7
Crnalic, 2011 [24]	2003–2008	Sweden	54	54	HR: 72 (54–88); HN: 77 (60–85)	C-1,CT-1,T-43,L-9	HR: 190 (0.5–5139); HN: 140 (21–3704)	6:2; 7:15; 8:7; 9:7; 10:3; NA:20	12	52	1:35; 2:17; 3–4:2	KPS (HR): 10–70:33; 80–100:8	1)HR: 5(range, 0–36)m; 2)OS%-1/3/6/12 m: 89/76/59/41	HR:41 HN:13	7
Drzymalski, 2010 [25]	1990–2009	USA	333	77	68 (43–90)	NA	58.2 (0–19,572)	≥7: 246	37	278	NA	NA	1)24 (95%CI,21–28)m; 2)OS%-12 m:73	NA	8
Crnalic, 2012 [14]	2003–2010	Sweden	68	68	HR: 71 (54–88); HN: 77 (60–88)	NA	HR:140 (0.06–5139); HN:147 (21–10,000)	≤6:3; 7:19; 8:9; 9:9; 10:3; NA: 25	17	NA	NA	KPS: 10–70:47; 80–100:21	1)NA 2)OS%-1,3,6,12,24 m: 89.7/72.1/57.4/44.1/23.5	HR:53; HN:15	7
Zakaria, 2018 [26]	2002–2012	USA	92	NA	73 (51–92)	NA	NA	NA	NA	NA	1:30; 2:24; 3–4:37	NA	1)4.1 (95%CI, 3.3–6.6)m; 2)NA	NA	6
Rades, 2015 [27]	NA	Germany	243	243	76	NA	NA	NA	65	156	1–2:94; 3–4:84; ≥5:65	ECOG: 1–2:107; 3–4:136	1)NA; 2)OS%-6/12 m:58.4/46.9	NA	6
Lehrmann-Lerche, 2019 [28]	2010–2011	Denmark	76	76	73.2 (50.6–95.8)	C-5, T-53, L-41, S-18	NA	≤7:14; 8:18; ≥9:32; NA:12	NA	48	1:19; 2:19; ≥3:38	NA	1)4.9 (95%CI, 3.6–6.2)m; 2)OS%-3/6/12 m: 64/42/21	NA	8
Weber, 2013 [29]	NA	Germany	95	95	NA	NA	NA	NA	69	NA	1–2:27; ≥3:68	ECOG: 1–2:32; 3–4:63	1)NA; 2)OS%-6/12 m:57.9/46.3	NA	7

*NA* Not available, *MSCC* Metastatic spinal cord compression, *OS%* Percentage of overall survival, *NOS* Newcastle-Ottawa Scale, *PSA* Prostate-specific antigen, *HR* Hormone-refractory prostate cancer, *HN* Hormone-naive prostate cancer, *KPS* Karnofsky performance score, *ECOG* Eastern Cooperative Oncology Group. Distribution of involved vertebrae: *C* Cervical, *CT* Cervicothoracic, *T* Thoracic, *TL* Thoracolumbar, *L* Lumbar, *LS* Lumbosacral; *S* Sacral

The therapeutic modality performed in each study is displayed in Table 2. Various treatments to the primary PCa were performed, mainly including prostatectomy, hormone therapy (either orchiectomy or treatment with gonadotropin-releasing hormone agonists), radiation therapy and chemotherapy. Regarding the major treatment, five studies were performed with various decompression surgeries with or without instrumentation procedures. Various radiation courses were applied in four studies without operative procedures, while both surgical treatment and radiotherapy were applied in two studies. Different adjuvant therapies were applied before the major therapy, mainly including narcotics for pain management, steroids, radiation therapy and bisphophonates (BP). Following the major therapy, radiotherapy, BP, narcotics, steroids and chemotherapy were applied as adjuvant therapy. In the five studies [14–16, 22, 24] associated with operative procedures, post-operative complications were recorded in 37.4% (83 of 222) of the patients.

**Table 2:** Summary of therapeutic modalities performed in the included studies

Study ID	Treatment of primary prostate cancer	Adjuvant therapies prior to major treatment	Major treatment	Adjuvant therapies after major treatment	Indication for surgery	Reoperation	Complications
Ju, 2013 [15]	1) radical prostatectomy (*n* = 8); 2) TURP (*n* = 2); 3) EBRT (*n* = 14); 4) hormone treatment (23 GnRH agonists/ 1 orchiectomy)(*n* = 24); 5) chemotherapy (*n* = 10)	1) narcotics for pain management (*n* = 25); 2) steroids (*n* = 25); 3) RT to spinal lesions (*n* = 25)	a total of 31 procedures: 1)PDC*(n* = 24), ADC(*n* = 3), APDC(*n* = 4); 2) decompressive laminectomy (*n* = 12); corpectomy /+laminectomy (*n* = 19); 3)instrumentation: anterior(*n* = 3), posterior(*n* = 14), anterior+posterior(*n* = 11)	1) RT (*n* = 14)	1) rapidly progressive neurological deterioration; 2) spinal mechanical instability	4 for SCC	16
Meng, 2016 [16]	NA	none	1) thoracic and lumbar metastases: PDC + pedicle screws and rods +titanium mesh (*n* = 10)/ cement-wire construct (*n* = 14); 2) cervical metastases: ADC (*n* = 1) /PDC(*n* = 3) /APDC(*n* = 1) + stable-angle plate osteosynthesis +pedicle screws	1)BP(*n* = 16)	1) rapidly progressive neurological deterioration; 2) pathologic fracture	NA	9
Huddart, 1997 [21]	NA	1)high-dose steroids(*n* = 47); 2) hormone therapy (*n* = 69)	1) RT (*n* = 57); 2) operation (*n* = 13)	none	–	–	NA
Williams, 2009 [22]	1) radical prostatectomy(*n* = 10); 2) EBRT (*n* = 24); 3) hormone treatment with LHRH agonists or orchiectomy (*n* = 44); 4) chemotherapy (*n* = 35)	1)narcotics(*n* = 40); 2)steroids(*n* = 28); 3) RT (*n* = 24)	a total of 47 procedures: 1)PDC(*n* = 19), ADC(*n* = 14), APDC(*n* = 14); 2)instrumentation: anterior (*n* = 10), posterior (*n* = 14), anterior +posterior (*n* = 7), PMMA (*n* = 12)	1) RT (*n* = 11); 2)narcotics; 3)steroids	1) intractable pain; 2) SCC; 3) progressive spinal involvement	3	15
Rades, 2012 [23]	NA	NA	1)short-course RT (*n* = 243); 2)longer-course RT(*n* = 193)	NA	–	–	NA
Crnalic, 2011 [24]	1) LHRH agonists (*n* = 34); 2) orchiectomy (*n* = 20); 3) radical prostatectomy (*n* = 1); 4) curative RT (*n* = 3); 5)antiandrogens(*n* = 29); 6) chemotherapy (*n* = 5)	1) high-dose steroids (*n* = 51); 2) RT to other site (*n* = 17); 3) RT to spinal site (*n* = 6); 4)BP(*n* = 3); 5) radioisotopes (*n* = 4); 6)low-dose prednisone (*n* = 11)	1) PDC (*n* = 29); 2)PDC + pedicle screws /+ hooks (n = 25)	1) RT (*n* = 35); 2) chemotherapy (*n* = 10); 3)BP(n = 8)	1) neurological defcit due to SCC	1 for subdural hematoma	19
Drzymalski, 2010 [25]	NA	NA	NA	NA	–	–	NA
Crnalic, 2012 [14]	1) LHRH agonists (*n* = 44); 2) orchiectomy (*n* = 24); 3) radical prostatectomy (*n* = 2); 4) curative radiation therapy (*n* = 7); 5) antiandrogens (*n* = 35); 6) chemotherapy (*n* = 10); 7) radioisotopes (*n* = 5)	1)high-dose steroids (*n* = 64); 2) radiotherapy to other metastases (*n* = 23); 3) zoledronic acid (*n* = 4); 4)low-dose prednisone (*n* = 11)	1)PDC(*n* = 42); 2)PDC+ pedicle screws /+ hooks(*n* = 26)	1) RT (*n* = 44)	1) neurological deficit	NA	24
Zakaria, 2018 [26]	NA	1) BP (*n* = 61); 2)anti-angiogenic drugs(*n* = 11)	1)SBRT(*n* = 92)	NA	–	–	NA
Rades, 2015 [27]	NA	NA	1)short-course RT (*n* = 144); 2)longer-course RT(*n* = 99)	NA	–	–	NA
Lehrmann-Lerche, 2019 [28]	1) castrationbased therapy (*n* = 76)	1)high-dose corticosteroids (*n* = 76)	1)RT(*n* = 72); 2) decompression surgery+RT (*n* = 4)	NA	–	–	NA
Weber, 2013 [29]	NA	NA	1) RT (*n* = 95)	NA	–	–	NA

*TURP* Transurethral resection of the prostate, *GnRH* Gonadotropin-releasing hormone, *PDC* Posterior decompression, *ADC* Anterior decompression, *APDC* Anterior-posterior decompression, *SCC* Spinal cord compression, *LHRH* Luteinizing hormone-releasing hormone, *PMMA* Polymethylmethacrylate, *RT* Radiation therapy, *BP* Bisphosphonates, *NA* Not available

Generally, the included studies were of a favorable methodological quality by NOS. The mean NOS was 7.3 ± 0.9 stars, and no study has a NOS score of less than 6.

### Results of qualitative synthesis

**Fig. 3:**
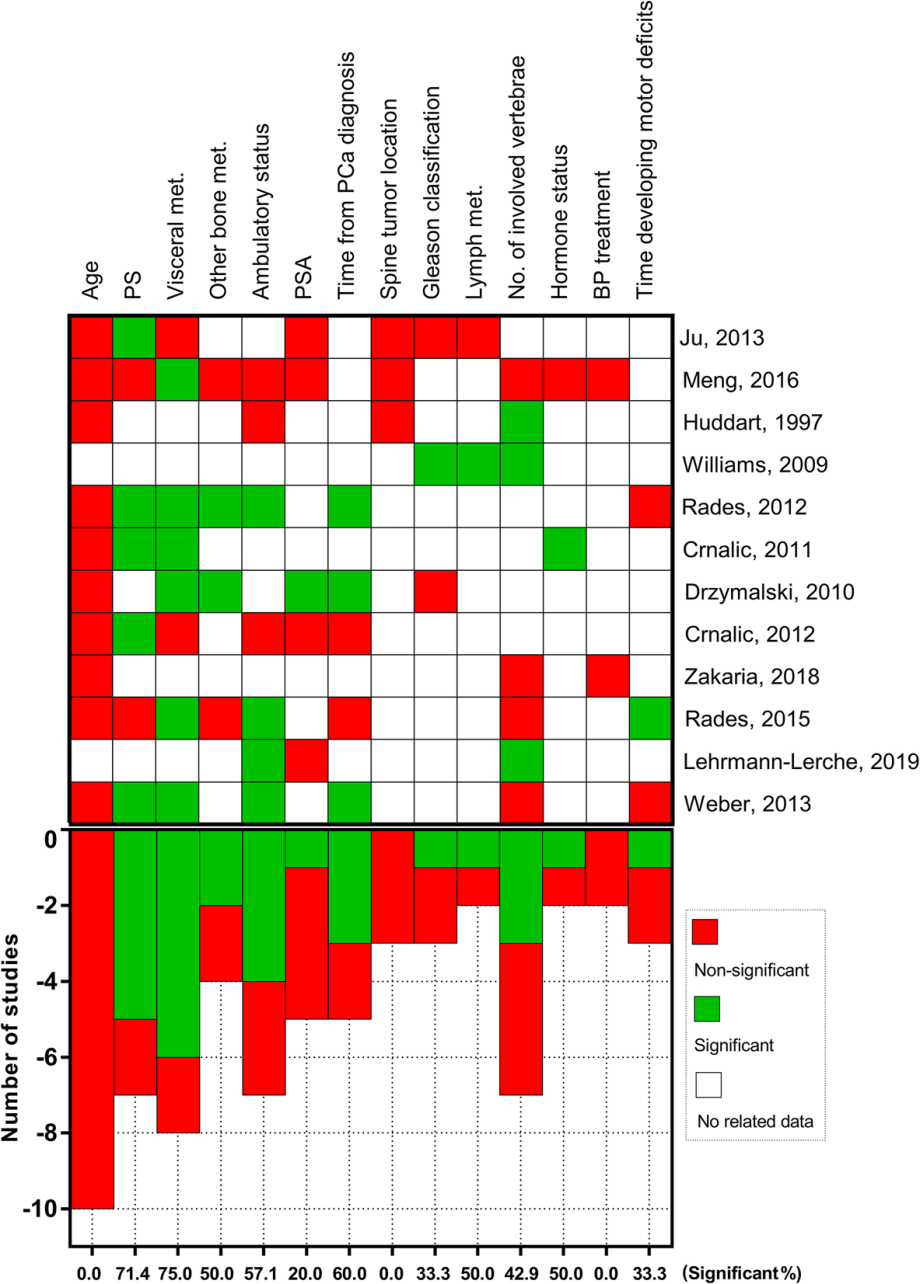
A plot depicting the significance of the available prognostic factors for predicting overall survival of patients with spinal metastasis from PCa. Generally, the overall survival was significantly associated with performance status, visceral metastasis, ambulatory status and time from PCa diagnosis in more than half of the available studies

As shown in Fig. 3, a total of 14 prognostic factors were assessed in at least two primary studies by either univariate or multivariate analysis. These factors include age at treatment, performance status (either Karnofsky performance score [KPS] or Eastern Cooperative Oncology Group-performance score [ECOG]), visceral metastasis, other bone metastasis, ambulatory status, PSA, time from the primary PCa diagnosis, spinal tumor location, Gleason grade, lymph metastasis, number of involved vertebrae, hormone status, BP treatment and the time developing motor deficit. The overall survival was significantly associated with performance status, visceral metastasis, ambulatory status and time from PCa diagnosis in more than half of the available studies.

Some other factors were additionally analyzed in only one of the studies, such as applying of pre-operative RT to the spine, total doses of RT applied to the spinal lesions (≤30Gy vs. >30Gy), presence of major complications, surgical approach (anterior vs. non-anterior), previous prostatectomy, Crnalic prostate score, urinary and bowel continence, Tomita score, revised Tokuhashi score, previous hormone therapy, haemoglobin (> 12 g/L vs. ≤12 g/L), post-operative ambulatory status, presence of MSCC, anti-angiogenic drugs and psoas muscle measurements. Among these, Tomita score, previous hormonotherapy, haemoglobin, post-operative ambulatory status and psoas muscle measurements were presented to be significant prognostic factors of overall survival.

### Results of quantitative meta-analyses

**Fig. 4:**
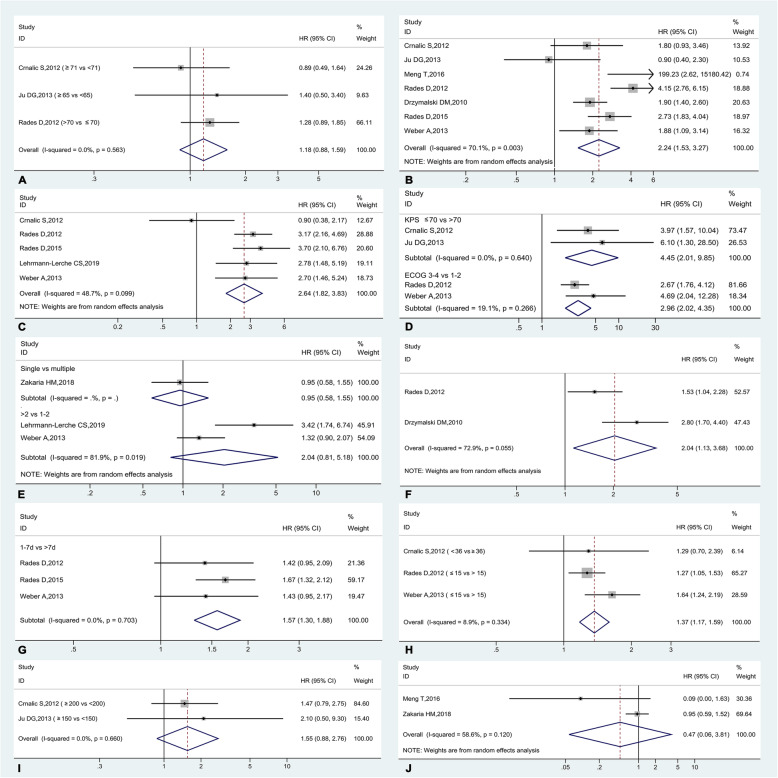
The forest plots for the prognostic factors with data available in two or more studies, including the age (**a**), visceral metastasis (**b**), ambulatory status (**c**), performance status (**d**), number of involved vertebrae (**e**), extraspinal bone metastasis (**f**), time developing motor deficit (**g**), time interval from primary PCa diagnosis (**h**), PSA level (**i**), and bisphosphonates treatment (**j**)

Figure 4a-h show the forest plots of all available predictors of survival, demonstrating that overall survival was significantly influenced by visceral metastasis (present vs. absent, HR = 2.24, 95%CI: 1.53–3.27, *p* < 0.001), pre-treatment ambulatory status (non-ambulatory vs. ambulatory, HR = 2.64, 95%CI: 1.82–3.83, *p* < 0.001), KPS (≤70 vs. > 70, HR = 4.45, 95%CI: 2.01–9.85, *p* < 0.001), ECOG (3–4 vs. 1–2, HR = 2.96, 95%CI: 2.02–4.35, *p* < 0.001), extraspinal bone metastasis (present vs. absent, HR = 2.04, 95%CI: 1.13–3.68, *p* = 0.018), time developing motor deficit (1–7 vs. > 7, HR = 1.57, 95%CI: 1.30–1.88, *p* < 0.001) and time from PCa diagnosis (HR = 1.37, 95%CI: 1.17–1.59, *p* < 0.001), while no significant prognostic effect on overall survival was found for age (HR = 1.18, 95%CI: 0.88–1.59, *p* = 0.269), number of involved vertebrae (single vs. multiple, HR = 0.95, 95%CI: 0.58–1.55, *p* = 0.839; > 2 vs. 1–2, HR = 2.04, 95%CI: 0.81–5.18, *p* = 0.130),PSA (HR = 1.55, 95%CI: 0.88–2.76, *p* = 0.129), and BP treatment (HR = 0.47, 95%CI: 0.06–3.81, *p* = 0.480). Random-effect model was selected for the syntheses of visceral metastasis, ambulatory status, number of involved vertebrae, other bone metastasis, and BP treatment due to significant heterogeneity.

### Sensitivity analysis and publication bias test

The results of sensitivity analysis and publication bias test for visceral metastasis and ambulatory status are available in Supplementary Fig. S1 and S2. No study was found to cause significant instability when it was omitted from the synthesis. No significant publication bias was found according to the methods of Egger’s and Begg’s tests.

## Discussion

In this study, we systematically reviewed the prognostic factors of overall survival in patients with metastatic spinal disease from PCa, and our quantitative meta-analyses demonstrated that presence of visceral metastasis, pre-treatment ambulatory status, KPS /ECOG, extraspinal bone metastasis, time developing motor deficit and time from PCa diagnosis were significant predictors for overall survival.

For guiding the treatment of spinal metastasis, many scoring systems have been established based on the significant prognostic factors [10–13]. Of these, the scoring systems of Tokuhashi [10] and Tomita [11] are the most commonly used tools for predicting overall survival. In these scores, however, various primary tumor types were included for developing the models, causing the low accuracy of prediction for some specific tumors due to that some special factors for different tumors were neglected, such as the PSA level, hormonotherapy and Gleason classification in spinal metastasis from PCa. Thus, several predicting scores have been developed for patients with metastatic spinal disease secondary to PCa [14, 23, 27]. In the study of Crnalic et al. [14], 68 consecutive patients operated for MSCC were used for survival analysis and new score development. As a result, four predictors including hormone status, KPS, visceral metastasis and PSA were significantly associated with the overall survival and listed in the scoring system. Rades et al. [27] developed a survival score for the elderly PCa patients with spinal metastases based on a total of 243 patients, and the ambulatory status, visceral metastasis and time developing motor deficits were the significant characteristics in the final score. However, the treatment related factors such as target therapy, immunotherapy and hormonotherapy, which has obviously changed the patients’ life expectancy, were seldom involved in these scores.

In our current systematic review and meta-analysis, we re-analyzed the prognostic effects of factors involved in 12 primary studies and 1566 patients. Among the identified significant factors, presence of visceral metastasis has been commonly accepted in most of the previous studies since that visceral metastasis may make the patients too deteriorated to tolerate some more invasive and curative therapies [10–12, 30]. Being similar to the effect of co-exist of visceral metastasis, presence of extraspinal metastasis represents a diffused status of the tumor and an increased tumor burden to patients [10, 11, 30]. Thus, extraspinal bone metastasis was accepted as a significant factor in many scoring systems like the Enkaoua [30], Tokuhashi [10] and Tomita [11], which was accordant with the result of the current study.

The neurological status is a relatively contradictory factor in predicting patients’ survival. It was not included as a prognostic factor in the scoring systems of Crnalic [14], Bauer [13], Bartels [31] and Tomita [11]. They believed that even in patients with neurological dysfunction, individualized treatment, such as decompression surgery, can greatly improve the neurological function of patients, thereby obtaining a relatively long survival period. Van der Linden et al. [12] also demonstrated that neurological status has no significant influence on the overall survival and they speculated that spinal cord paralysis could only reflect the anatomical location or size of spinal metastases. However, pre-treatment neurological status was included in the scoring systems of Tokuhashi [10], Sioutos [32], and Enkaoua [30]. In general, they speculated that patients with walking dysfunction due to MSCC are more prone to suffer from some fatal complications, such as pneumonia (especially in cancer patients with immunosuppression) [33]. In our study, we also found a 2.64-fold increase on the modality rate in non-ambulatory patients when compared to the ambulatory patients.

Performance status, mainly including KPS and ECOG, is used to assess the patient’s overall health and functional status. Patients with better performance status could better tolerate the side effects of some invasive anti-tumor treatments. In the study of Crnalic et al. [14], patients with KPS of less than 80 showed a 3.97-fold increase on the overall risk of modality than those with KPS of 80–100, and the KPS was included as a significant predictor in the score. The significant prognostic effects of the time developing motor deficit and time interval from primary PCa diagnosis are also in accordance with other previous studies.

The PSA level, as a PCa-specific factor which could partly reflect the degree of tumor progress, was analyzed in five studies [14–16, 25, 28] but only significant in one study [25]. In the study of Crnalic et al. [14], multivariate analysis showed that serum PSA concentration had no statistically significant effect on overall survival in patients with prostate spinal metastases. Despite this, PSA level was still included in their scoring system as they believed that higher serum PSA concentrations can reflect the advanced state of PCa. BP treatment, which was commonly used as a adjuvant therapy to inhibit the bony destruction, was also proven to be non-significant on the influence to overall survival.

In order to guide the incorporation of multi-disciplinary treatment for spinal metastatic tumors, the neurologic, oncologic, mechanical, and systemic (NOMS) framework was proposed by Laufer et al. [34], emphasizing the neurologic, oncologic, mechanical stability and systematic considerations. It is crucial to consider the patients’ ability to tolerate the treatment procedures based on the systematic status or overall survival, which could be easily assessed through pre-treatment characteristics. Thus, all of the potential predictors associated with overall survival reviewed and analyzed in this study should be take into consideration when selecting a treatment modality for spinal metastasis secondary to PCa.

### Limitation

This study, nevertheless, has some limitations. Firstly, the primary studies included were all retrospective cohort studies, which may associated with inherent risk of bias in the data collection. Then, most of the studies were focused on assessing the prognostic effects of patients’ characteristics, but the treatments applied were different among the studies, which may have influence to the overall survival. Finally, several factors were only analyzed in one of the studies, and the quantitative syntheses for these factors were not applicable. Thus, some more primary cohort studies may be needed to further identify the prognostic effects of these factors.

## Conclusions

This study identified all available prognostic factors for spinal metastasis secondary to PCa based on published cohort studies. According to the results of meta-analyses, presence of visceral metastasis, ambulatory status, extraspinal bone metastasis, performance status, time developing motor deficit and time interval from primary tumor diagnosis were demonstrated to be significantly associated with the overall survival. When selecting the treatment modality for patients with spinal metastasis from PCa, the clinicians should fully consider the patients’ systematic status based on all associated prognostic factors.

## Supplementary information

**Additional file 1 Supplementary Figure S1.** Results of sensitivity analysis for visceral metastasis (A) and ambulatory status (B). No study was found to cause significant instability when it was omitted from the synthesis.

**Additional file 2 Supplementary Figure S2.** Results of publication bias test for visceral metastasis (A&B) and ambulatory status (C&D). No significant publication bias was found according to the methods of Egger’s (*p* > 0.100) and Begg’s tests (*p* > 0.050).

## Data Availability

The authors declare that all the data supporting the findings of this study are available within the article and its supplementary information files.

## Abbreviations

PCa: Prostate cancer
HR: Hazard ratio
KPS: Karnofsky performance score
ECOG: Eastern Cooperative Oncology Group
95%CI: 95% confidence interval
MSCC: Metastatic spinal cord compression
PSA: Prostate-specific antigen
NOS: Newcastle-Ottawa Scale
BP: Bisphophonates
RT: Radiation therapy
