# Supplementary figures and images for: Prognostic factors for overall survival in patients with spinal metastasis secondary to prostate cancer: a systematic review and meta-analysis
Source: BMC Musculoskelet Disord. 2020 Jun 17;21:388. doi: 10.1186/s12891-020-03412-0 (PMC7298793; doi:10.1186/s12891-020-03412-0)

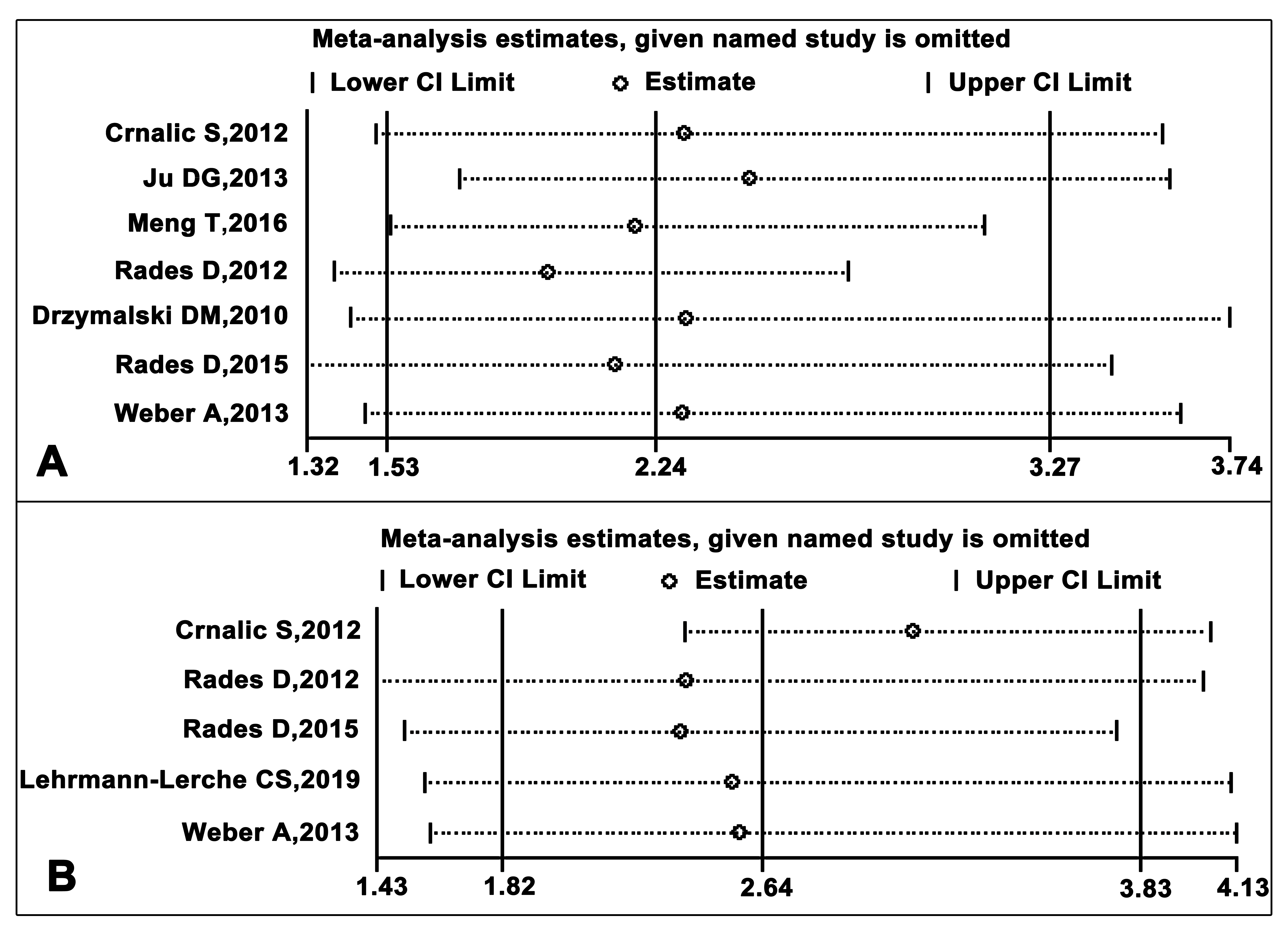

Supplement: Supplementary file 1 — Additional file 1 Supplementary Figure S1. Results of sensitivity analysis for visceral metastasis (A) and ambulatory status (B). No study was found to cause significant instability when it was omitted from the synthesis. [file 12891_2020_3412_MOESM1_ESM.tif]

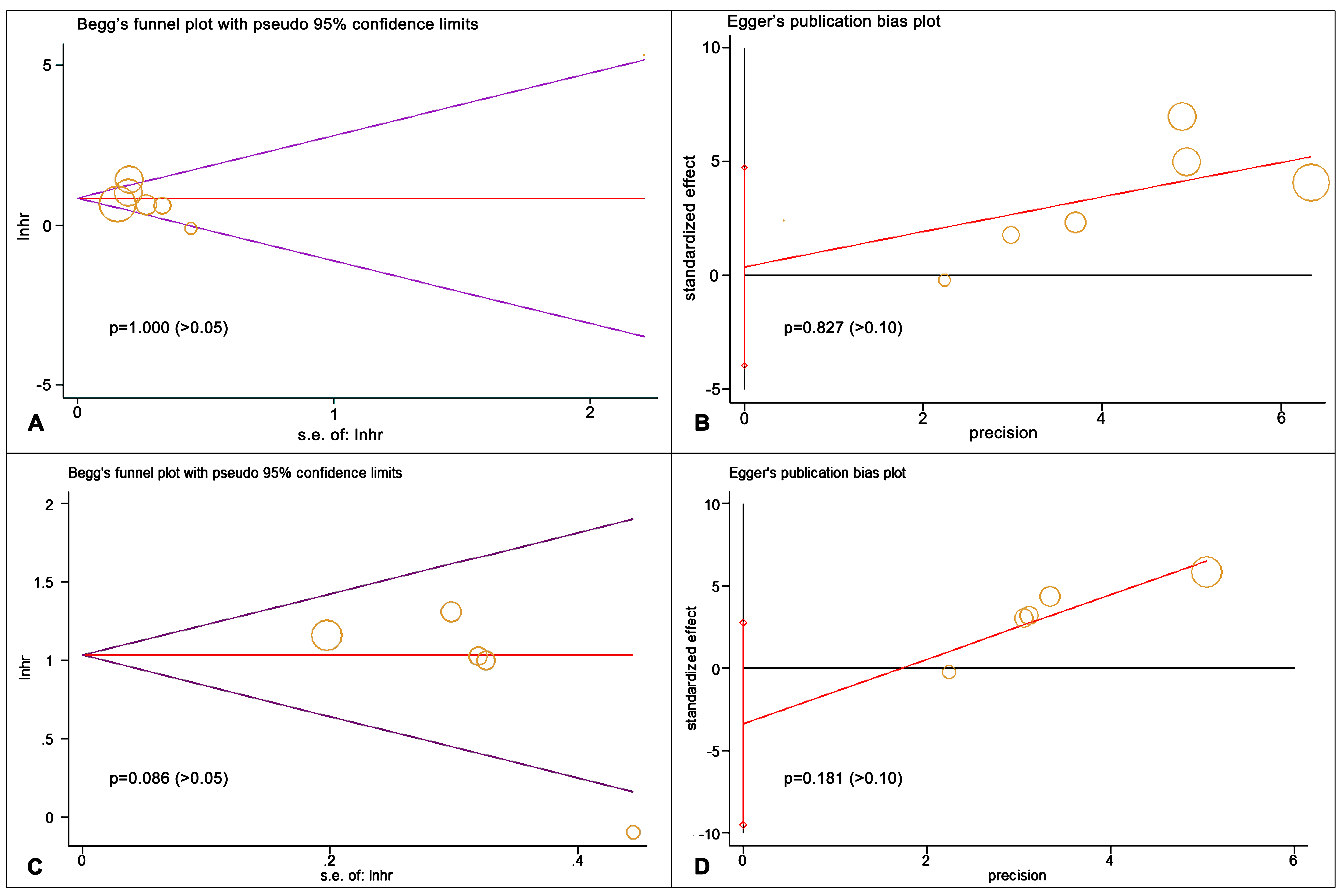

Supplement: Supplementary file 2 — Additional file 2 Supplementary Figure S2. Results of publication bias test for visceral metastasis (A&B) and ambulatory status (C&D). No significant publication bias was found according to the methods of Egger’s (p > 0.100) and Begg’s tests (p > 0.050). [file 12891_2020_3412_MOESM2_ESM.tif]
